# Isotopic discrimination in helminths infecting coral reef fishes depends on parasite group, habitat within host, and host stable isotope value

**DOI:** 10.1038/s41598-021-84255-0

**Published:** 2021-02-25

**Authors:** Philip M. Riekenberg, Marine J. Briand, Thibaud Moléana, Pierre Sasal, Marcel T. J. van der Meer, David W. Thieltges, Yves Letourneur

**Affiliations:** 1 Department of Marine Microbiology & Biogeochemistry, NIOZ Royal Netherlands Institute for Sea Research, PO Box 59, 1790AB Den Hoorn, The Netherlands; 2 UMR Entropie (Université de La Réunion, IRD, CNRS, IFREMER, Université de la Nouvelle-Calédonie), Université de la Nouvelle-Calédonie, BP R4, 98851 Nouméa Cedex, New Caledonia; 3 EPHE-UPVD-CNRS USR CNRS 3278, Centre de Recherche Insulaire et Observatoire de l’Environnement (CRIOBE), PSL Research University, Université de Perpignan Via Domitia, 58 avenue Paul Alduy, 66860 Perpignan, France; 4 Department of Coastal Systems, NIOZ Royal Netherlands Institute for Sea Research, PO Box 59, 1790 AB Den Hoorn, Texel, The Netherlands; 5 Laboratoire d’Excellence “CORAIL”, Perpignan, France

**Keywords:** Ecosystem ecology, Stable isotope analysis

## Abstract

Stable isotopes of carbon and nitrogen characterize trophic relationships in predator–prey relationships, with clear differences between consumer and diet (discrimination factor Δ^13^C and Δ^15^N). However, parasite–host isotopic relationships remain unclear, with Δ^13^C and Δ^15^N remaining incompletely characterized, especially for helminths. In this study, we used stable isotopes to determine discrimination factors for 13 parasite–host pairings of helminths in coral reef fish. Differences in Δ^15^N values grouped according to parasite groups and habitat within the host with positive Δ^15^N values observed for trematodes and nematodes from the digestive tract and variable Δ^15^N values observed for cestodes and nematodes from the general cavity. Furthermore, Δ^13^C values showed more complex patterns with no effect of parasite group or habitat within host. A negative relationship was observed between Δ^15^N and host δ^15^N values among different host-parasite pairings as well as within 7 out of the 13 pairings, indicating that host metabolic processing affects host-parasite discrimination values. In contrast, no relationships were observed for Δ^13^C values. Our results indicate that parasite group, habitat within host, and host stable isotope value drive Δ^15^N of helminths in coral reef fish while their effect on Δ^13^C is more idiosyncratic. These results call for use of taxon- or species-specific and scaled framework for bulk stable isotopes in the trophic ecology of parasites.

## Introduction

Parasitism is a common life strategy for consumers and is ubiquitous amongst food webs^1^. The role of parasites in aquatic food chains has been shown to be fundamental^2,3^ and the inclusion of parasitic relationships within food webs dramatically increases the number of trophic links within ecosystems^4^. Despite this, host-parasite relationships remain a neglected component during the evaluation of biodiversity^5^, especially for systems with high biodiversity such as coral reefs^6^. Trophic relationships for parasites^7^ remain poorly characterized within food webs as small size, multidisciplinary requirements for identification, and cryptic lifestyles (e.g. multiple hosts associated to multiple larval stages) make identification and characterization of these relationships difficult.

Stable isotope techniques are routinely utilized to study trophic relationships within food webs^8,9^ by using trophic discrimination factors for carbon and nitrogen (Δ^13^C and Δ^15^N) to account for the stepwise increase in δ^13^C and δ^15^N (‰) that occurs between diet and consumer during metabolism^10–12^. However, parasites do not generally follow this relationship with parasite–host discrimination factors (Δ^13^C or Δ^15^N) observed ranging from considerably higher than typical trophic discrimination between predator and prey to negative values for Δ^13^C or Δ^15^N across a variety of taxa^1,13,14^.

Amongst helminths in fish hosts, cestodes and nematodes are usually depleted in δ^15^N values *versus* their hosts^13,15–17^ and vary in Δ^13^C, while trematodes have been found to vary in both Δ^13^C and Δ^15^N^18,19^. However, there can also be considerable variation within these helminth groups^14,20^ and within other coral reef parasites such as copepods, cymothoids, gnathiids, isopods, and monogeneans^2,21–23^. Distinct differences for trophic discrimination in parasitic relationships are potentially caused by the combined effects of unique feeding ecologies, often reduced metabolic capabilities of the parasitic taxa being investigated^24,25^, and host metabolic effects due to parasitism^26^. Feeding ecology varies depending on whether the parasite feeds upon host tissue exclusively (on host or within; tissue type dependent^18^), is able to supplement with material from within the dietary tract as the host feeds or from the environment (e.g. prey items, detritus, mucus, or blood^27^), or can directly uptake nutrients from host tissues^28^. In addition, it has been suggested that trophic discrimination factors of parasites may not be fixed but scale with the isotopic signature of their hosts, both within^29^ and among parasite species^14^.

Despite multiple investigations, clear stable isotope discrimination patterns between major parasite groups have not emerged, making simple incorporation of parasites into food web studies using a single universal trophic discrimination factor impossible. This knowledge gap warrants further investigation into the drivers of discrimination factors in helminths. In this study, we examined both δ^13^C and δ^15^N values from whole tissue of 136 helminth parasite–host pairings from coral reef fish to determine the isotopic relationship between taxonomically distinct groups of helminth parasites and to investigate the effect of the habitat within the host and host isotopic value on helminth isotopic discrimination. We expected that parasite isotopic enrichments and variability *versus* their host will be larger in parasites located in the dietary tract as their diet may not only include host material while parasites in the body cavity that solely utilize host tissues will have less variability in their isotopic discrimination. In addition, we expect a negative scaling of parasite discrimination factors with the δ^13^C and δ^15^N values of their hosts.

## Results

**Figure 1:**
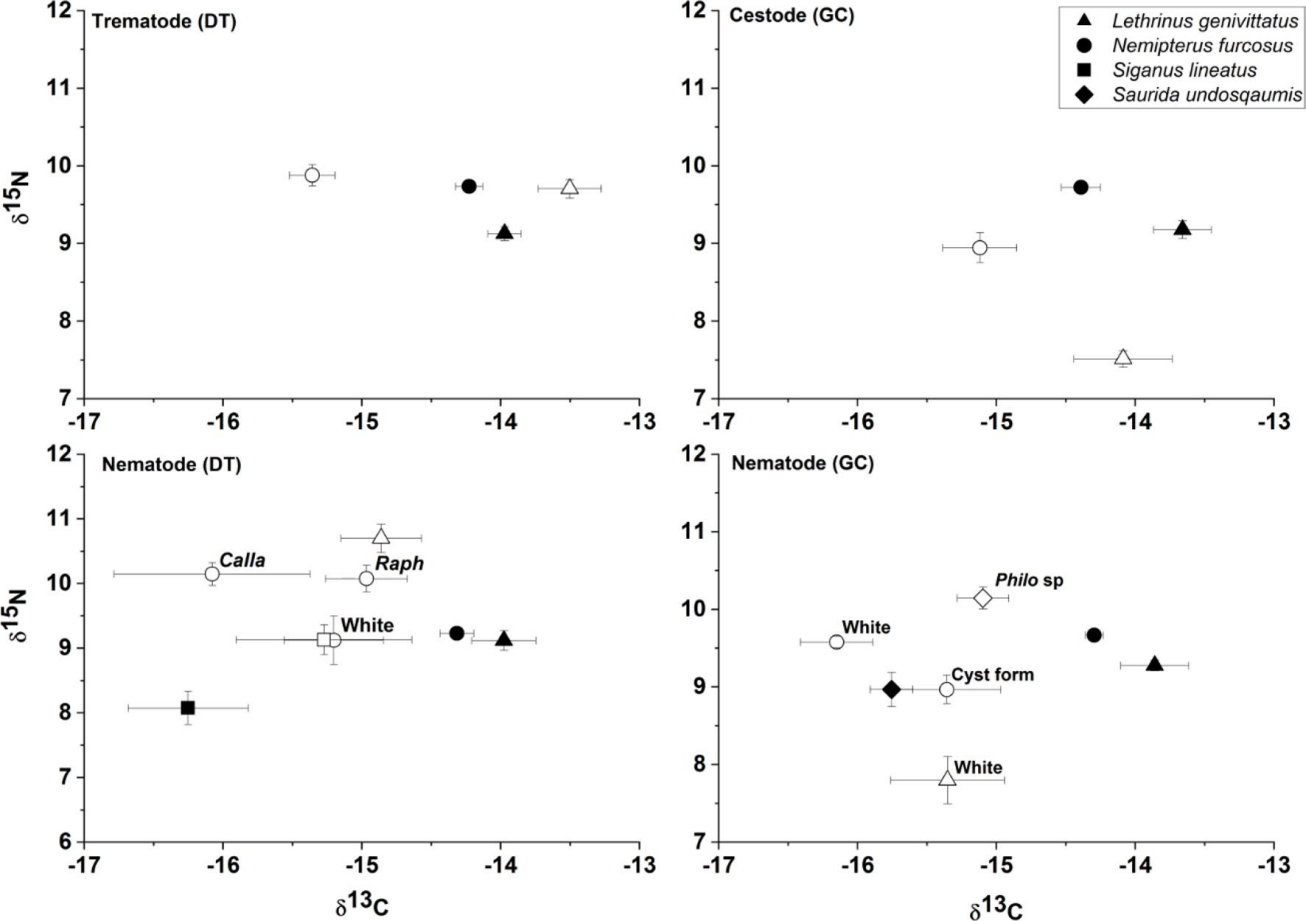
Mean δ^15^N and δ^13^C composition (± SD) for parasite and reef fish host pairings. Parasites and hosts are displayed with open and filled symbols, respectively. Dietary tract (DT) and general cavity (GC) refer to the parasite habitat within fish hosts.

**Figure 2:**
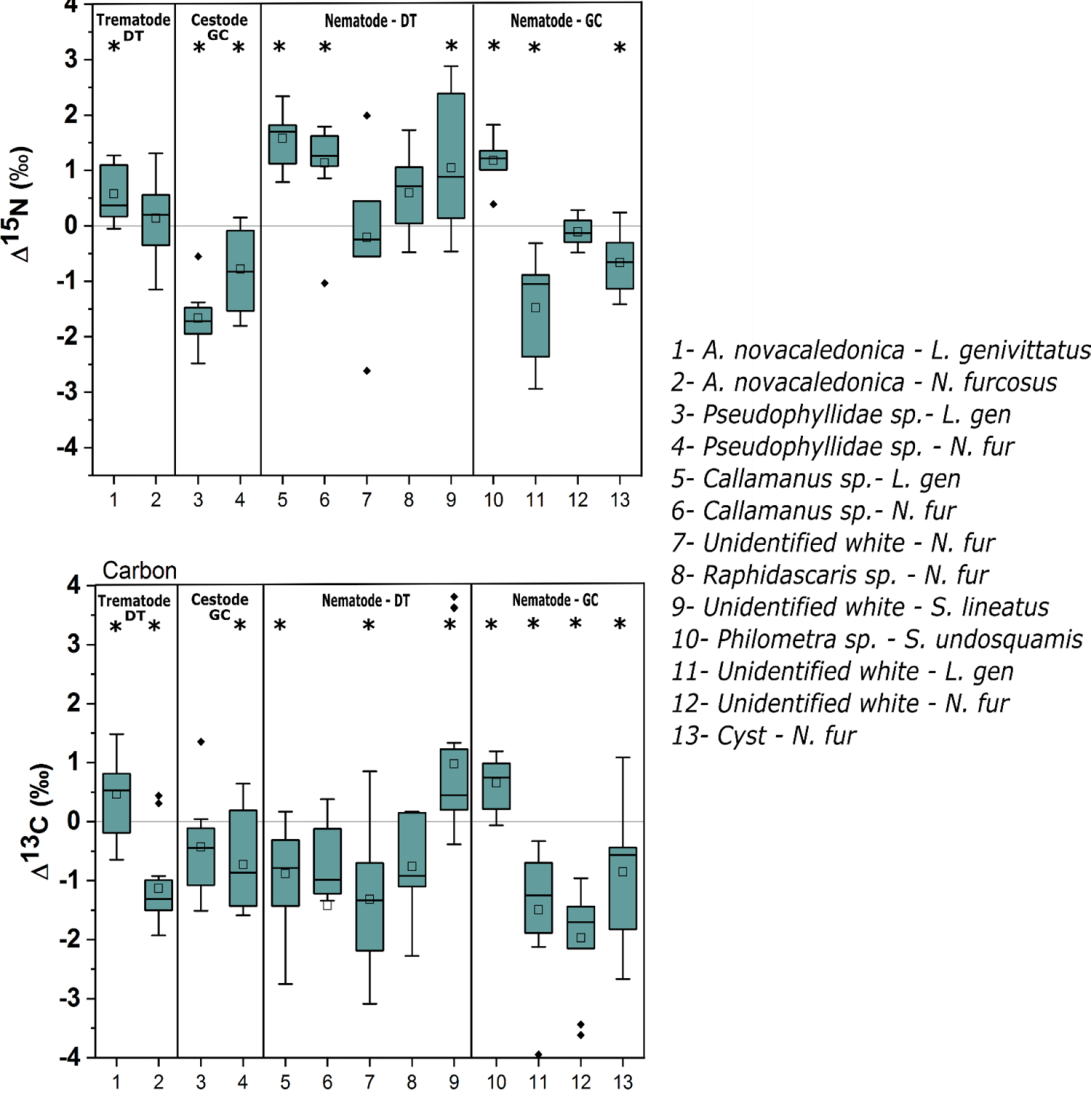
Δ^15^N and Δ^13^C values (‰) for parasite–host couplings examined in this study. Asterisks indicate significant differences between host and parasite at an α = 0.05. Dietary tract (DT) and general cavity (GC) refer to the parasite habitat within fish hosts. For the boxplots, squares indicate mean, lines indicate median, boxes indicate upper and lower quartiles, and whiskers indicate 1.5 quartile ranges. Black diamonds are outliers.

**Figure 3:**
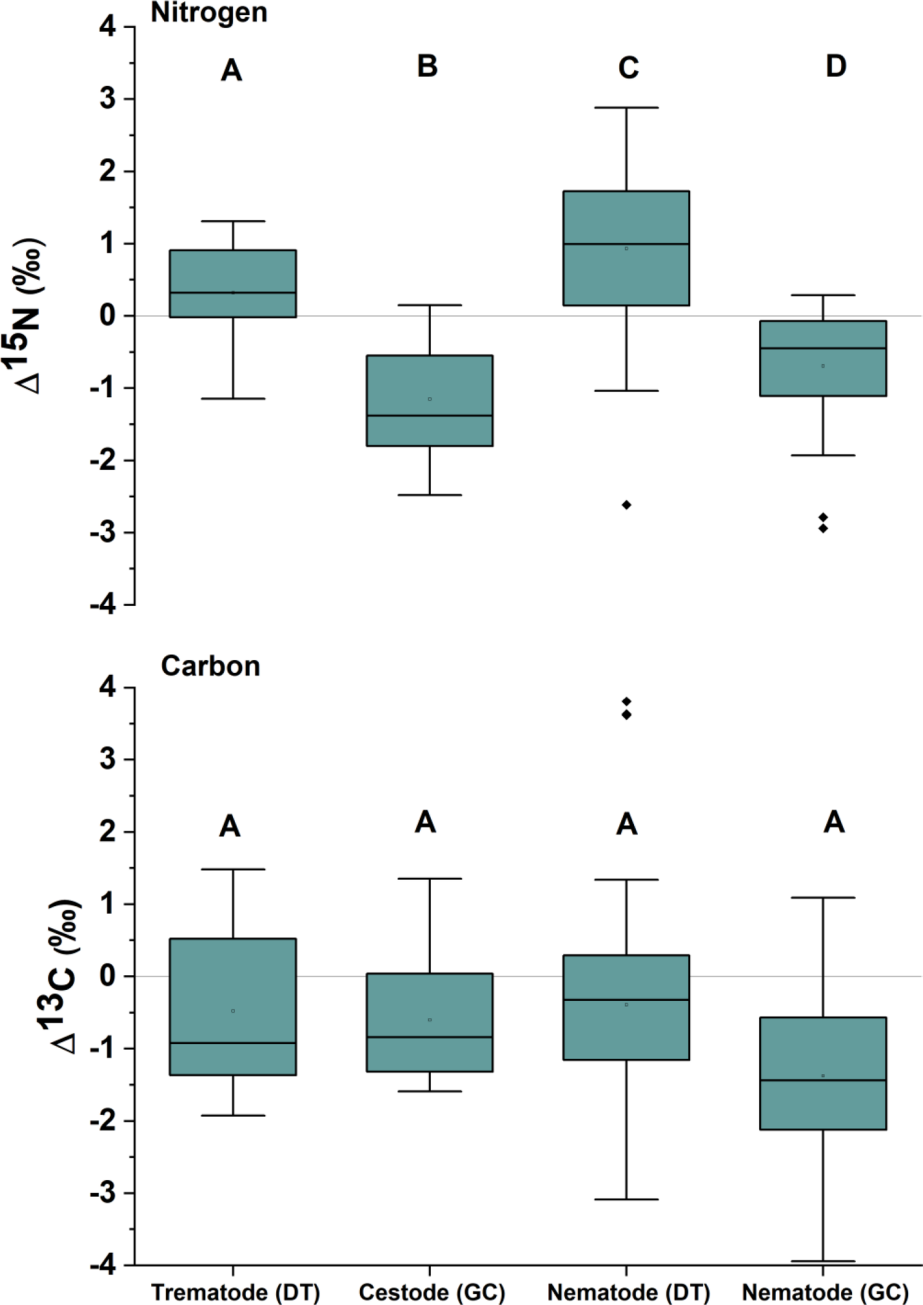
Δ^15^N and Δ^13^C values (‰) for trematodes and cestodes, and nematodes separated by parasite habitat within host. Letters indicate significant differences (post hoc Tukey’s test, α = 0.05). Dietary tract (DT) and general cavity (GC) refer to the parasite habitat within fish hosts. For the boxplots, black dots indicate mean, lines indicate median, boxes indicate upper and lower quartiles, and whiskers indicate 1.5 quartile ranges. Black diamonds are outliers.

**Figure 4:**
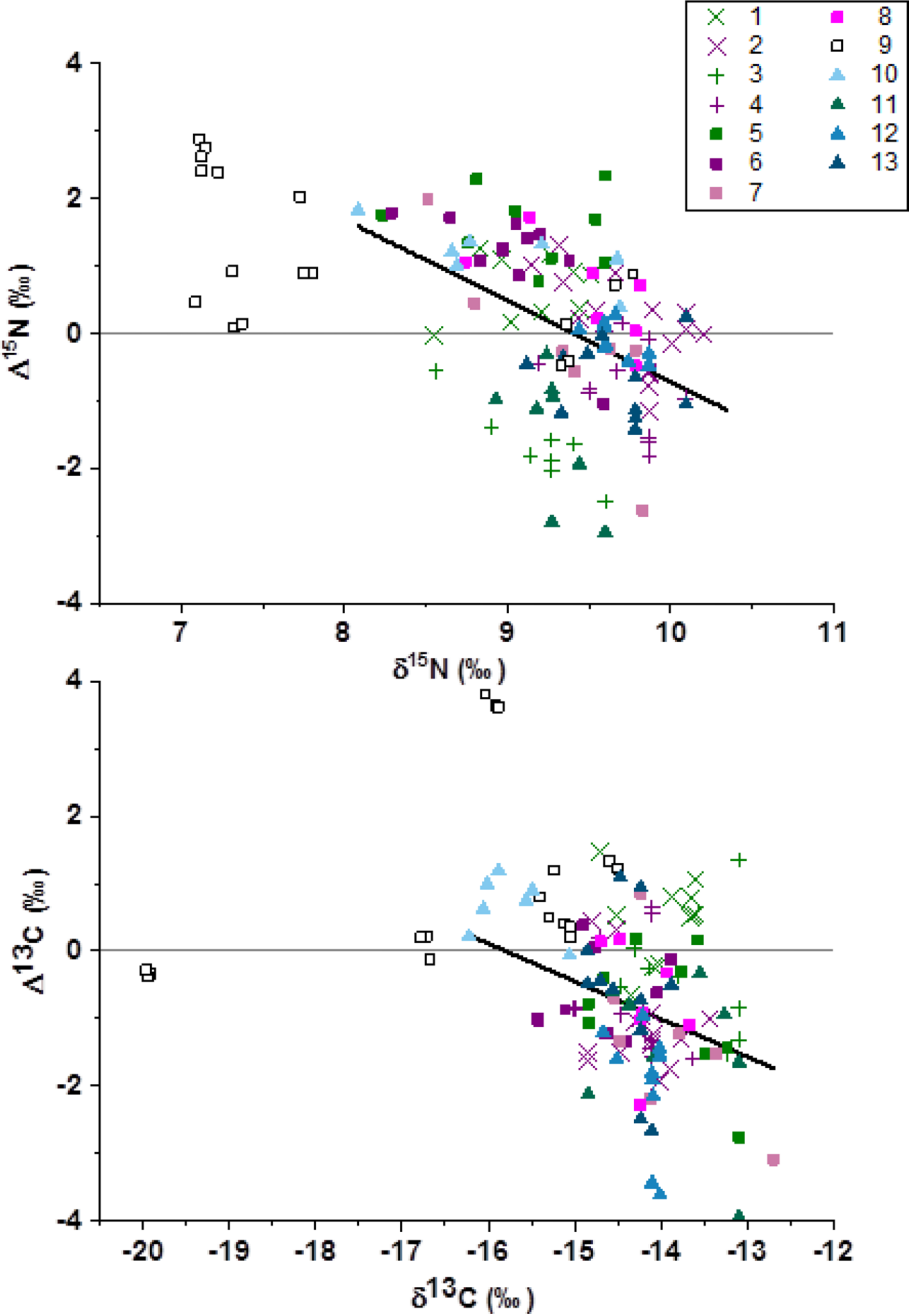
Host δ^15^N values versus Δ^15^N values classified for each individual parasite–host pairing. Labeling follows the pairing order 1–13 from Fig. 2 with X indicating trematodes, + indicating cestodes, squares indicating dietary tract nematodes and triangles indicating general cavity nematodes. Regression lines indicate the significant relationships for the combined pairings from the three predatory (invertivore or piscivore) fish in the dataset (i.e. excluding samples from the herbivore host *S. lineatus* (open squares, 9)) for nitrogen (slope = − 1.2, R^2^ = 0.071, *p* < 0.001) and carbon (slope = − 0.56, R^2^ = 0.196, *p* = 0.17).

We examined the isotopic discrimination of 136 helminth parasite–host pairings including trematodes (n = 27), cestodes (n = 19), and nematodes (n = 90) from 4 host reef lagoon-associated fish species (from the families Lethrinidae, Nemipteridae, Siganidae and Synodontidae; Table 1, Fig. 1). One trematode species and five of the nematode species were sampled from the dietary tracts (DT) of host species while the remaining one cestode and 4 nematode species were samples from the general cavity (GC). Six out of the 13 Δ^15^N values for parasite–host pairs were negative (Supplementary Table 1), with positive relationships (1.05 to 1.58‰) predominately occurring in dietary tract associated parasites (Fig. 1). In 9 out of 13 cases, δ^15^N values were significantly different between parasites and hosts (Supplemental Table 1 and Fig. 2). For the three parasite pairs found in both *Lethrinus genivittatus* and *Nemipterus furcosus* (i.e. *Allardia novacaledonica, Callamanus* sp., and Pseudophyllidae), Δ^13^C and Δ^15^N were consistently the same between the parasite–host pairings within the same host, either positive or negative, except for carbon in *A. novacaledonica* (0.47‰ *L. genivitattus versus* -1.10‰ *N. furcosus*) (Supplemental Table 1, Fig. 2). For Δ^15^N values, we found significant differences between trematodes, cestodes and the two habitats (DT & GC) of nematodes within hosts (One-way ANOVA: F_3, 132_:26.9 *p* < 0.001; Fig. 3). Host δ^15^N was examined *versus* Δ^15^N and we found that a linear regression for all of the predatory host pairings (e.g. with the herbivore host pairing white nematode—*Siganus lineatus* removed) had a negative slope of − 1.2 (R^2^ = 0.071; Fig. 4) with negative slopes observed within individual pairings that were different than 0 for 7 of the parasite–host pairings at α = 0.05 (Fig. 4; Supplementary Table 2).

**Table 1:** Number (N) of parasite–host pairings analyzed from different habitats within hosts, the digestive tract (DT) or the general cavity (GC), from four fish hosts sampled in the coral reef lagoon of New Caledonia.

Fish host (size range, cm; trophic level; family; feeding style)	Parasite	Attachment site on host (DT/GC)	N
*Lethrinus genivittatus* (11.0–21.5; 2.9; Lethrinidae; invertivore)	*Adlardia novacaledonica* (Trematode)	DT	11
	Pseudophyllidae (Cestode)	GC	8
	*Callamanus* sp. (Nematode)	DT	9
	Unidentified white nematode	GC	8
*Nemipterus furcosus* (12.1–25.0; 3; Nemipteridae; invertivore)	*Aldardia novacaledonica* (Trematode)	DT	16
	Pseudophylidae (Cestode)	GC	11
	*Callamanus* sp. (Nematode)	DT	11
	*Rhaphidascaris* sp. (Nematode)	DT	7
	Unidentified white/cream nematode	GC	10
	Unidentified nematode (cyst form)	GC	13
	Unidentified white nematode	DT	7
*Siganus lineatus* (17.8–25.6; 2.5; Siganidae; herbivore)	*Philometra* sp. (Nematode)	GC	18
*Saurida undosquamis* (28.1–38.0; 2.8; Synodontidae; piscivore)	Unidentified white nematode	DT	7

Δ^13^C values of the pairings were generally negative with lower δ^13^C in the parasite than those of the host fish, except for *Philometra* sp./*Saurida undosquamis* (Δ^13^C − 0.06 to 1.19‰), white nematode/*S. lineatus* (Δ^13^C − 0.38 to 3.81‰) and *A. novacaledonica* / *L. genivittatus* (Δ^13^C 0.47 to 0.98‰, Figs. 2 and 3). The first two of these relationships occur for parasites in the digestive tract and the last one in the general cavity of the host fish. Among the 13 parasite–host pairings tested, significant differences between host and parasite δ^13^C were observed for 10 cases with a Δ^13^C range from − 0.73 to − 1.97‰. There was no clear Δ^13^C distinction observed between parasites found in the digestive tract *versus* those found in the general cavity. For Δ^13^C values, we found no significant differences between trematodes, cestodes and the two habitats (DT and GC) of nematodes within hosts (One-way ANOVA: F_3, 132_:1.4 *p* = 0.2; Fig. 3). Host δ^13^C was examined *versus* Δ^13^C and found that a linear regression for all of the pairings combined minus the herbivore pairing had a negative slope of − 0.56 (R^2^ = 0.196, *p* < 0.001; Fig. 4), but that linear regressions for individual pairings indicated no significant difference from 0 for those relationships (Supplementary Table 2).

**Figure 5:**
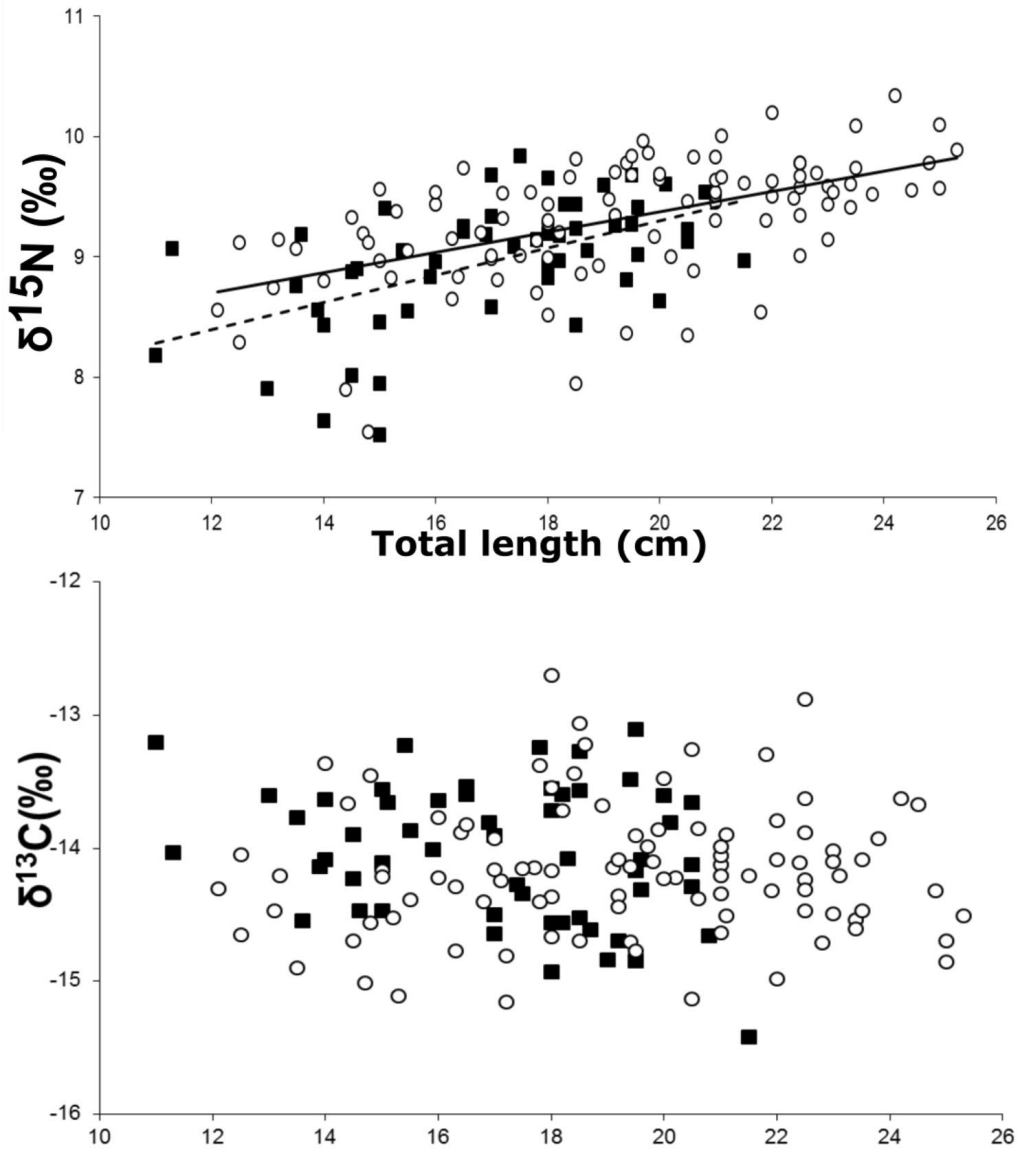
Relationship between fish total length (cm) and δ^15^N (‰) and δ^13^C (‰). Pearson correlations indicated significant increase in δ^15^N values for both *L. genivittatus* (solid squares, dashed line, R^2^ = 0.30, *p* < 0.001*)* and *N. furcosus* (open circles, solid line, R^2^ = 0.31, *p* < 0.001*)* with no corresponding increase for δ^13^C values.

Mean δ^13^C and δ^15^N values for the host species *L. genivittus* were − 14.04 ± 0.51‰ and 8.97 ± 0.52‰ (mean ± SD; Δ^13^C then Δ^15^N throughout), *N. furcosus* were − 14.16 ± 0.49‰ and 9.31 ± 0.50‰*, S. undosquamis* were − 16.25 ± 0.49‰ and 8.07 ± 1.09‰*,* and *S. lineatus* were − 15.75 ± 0.40‰ and 8.97 ± 0.59‰*,* respectively (Fig. 1; Supplementary Table 1), with significant differences observed between species for both δ^13^C and δ^15^N values (One-way ANOVA: δ^13^C, *F*_3, 177_:52.6, *p* < 0.001; δ^15^N, *F*_3, 177_:22.8, *p* < 0.001). Body size did not influence δ^13^C and δ^15^N values for *S. undosquamis* and *S. lineatus* for the size range and number of individuals considered here (Pearson correlation, p > 0.05 in all cases). By contrast, fish size significantly influenced δ^15^N values for *L. genivittus* and *N. furcosus* (p < 0.001 for both species; Fig. 5) but not for δ^13^C values (p > 0.05). Calculated trophic levels (Table 1) were similar for the whole populations of fish sampled in this study for *L. genivittus* and *S. undosquamis* (2.9 and 2.8, respectively), *S. lineatus* having the lowest, and *N. furcosus* having the highest trophic levels (2.5 and 3.0, respectively; One-way ANOVA: F_3, 137_:42.7, *p* < 0.001). Trophic levels were also higher for larger fish for both *L. genivattus* (2.6 and 2.9 for 11–15 cm and 18–21.5 cm individuals, respectively; One-way ANOVA: F_1, 39_:35.9, *p* < 0.001) and *N. furcosus* (2.8 and 3.0 for 12.1–15 cm and 20–25.3 cm individuals, respectively; One-way ANOVA: F_1, 61_:27.6, *p* < 0.001).

## Discussion

Δ^15^N varied inconsistently between and within taxa, with the most consistent result being elevated Δ^15^N (> 0‰) for dietary tract associated nematodes, likely associated with feeding on host dietary items in addition to tissue. Δ^13^C was consistently negative between parasite taxa and likely indicates increased reliance on fatty acids from the host to support tissue growth in reef fish-associated helminths. The varied relationships amongst and between taxa provide further evidence that parasite–host pairings are distinctly different than typical trophic relationships and warrant further investigation to adequately characterize parasite contributions to food webs.

### Δ^15^N differences for parasite–host pairings

Δ^15^N values showed no difference or were positive for the dietary tract associated trematode and nematodes (*Allardia, Callamanus, Rhaphidascaris,* and unidentified white) while the cestodes had negative values for both pairings examined. Δ^15^N values for the general cavity-associated nematodes varied, with a strong positive value for the gonad-associated *Philometra* sp.–*S. undosquamis* pairing, no difference observed for unidentified white nematode—*N. furcosus* pairing, and strong negative relationships for both the nematode cyst-type—*N. furcosus* and the unidentified white nematode—*L. genivittatus* pairings (Fig. 2).

Trematodes have been found to have positive to neutral Δ^15^N values regardless of infection site (e.g. dietary tract or general cavity^18,19^) indicating utilization of host-metabolized nitrogen derived from tissue in addition to nitrogen compounds derived from the host diet. The low Δ^15^N values (0.18 to 0.6‰) observed in this study demonstrate close association of the trematode with the host diet. Sole reliance on host tissue and therefore host-metabolized N would be expected to yield a “typical” trophic enrichment of ~ 3.4‰ expected between diet and consumer^8,11^, which is considerably larger than the observed Δ^15^N. Small Δ^15^N values likely reflect combined utilization of more ^15^N enriched sources of nitrogen derived from host tissue as well as more ^15^N depleted compounds either metabolized or directly assimilated. Negative Δ^15^N values have been previously observed for cestodes within fish hosts^15,16,18,19,27,30^ and may be caused by direct utilization of relatively ^15^N-depleted compounds from the host diet^16^ or metabolically recycled ^15^N-depleted amino acids produced by the gut microbial community^31^ that both cestodes and trematodes are well-positioned to utilize while residing in the dietary tract.

Similarly, three of the general cavity associated nematodes displayed negative or neutral Δ^15^N values, indicating that direct uptake from the host without further metabolic processing by the parasite of nitrogen compounds is a likely pathway for N in this taxa. This uptake was not consistent across the taxa, with different species that target similar infection sites (e.g. dietary tract *versus* general cavity) displaying considerably different Δ^15^N values. The single gonad-associated nematode (*Philometra* sp.) examined within *S. undosquamis* had a positive Δ^15^N indicating at least partial reliance on direct utilization of metabolized N from host tissues. The dietary tract-associated nematodes were generally ^15^N-enriched in comparison to their hosts indicating at least partial utilization of host-metabolized compounds from tissue or the taxa-dependent ability for nematodes to biosynthesize amino acids from nitrogenous compounds^25^.

### Δ^13^C differences for parasite–host pairings

Δ^13^C values were predominately neutral or negative for ten of the parasite–host pairings examined, with positive Δ^13^C only observed for the *A. novacaledonica—L. genivittatus*, white nematode—*S. lineatus*, and *Philometra* sp.—*S. undosquamis* pairings. No change or depletion in δ^13^C does not agree with the expected 0.5–1‰ increase that is usually expected for trophic interactions^11^, but likely reflects reliance on lipids and fatty acids directly derived from the host or host diet to support helminth tissue growth. Platyhelminthes and some nematodes have been found to be incapable of de novo fatty acid synthesis and to have to rely on fatty acids derived from the host^25,32^ due to incomplete metabolic pathways for lipid biosynthesis. Direct uptake of fatty acids and other lipids from the host would be expected to coincide with a minimum carbon fractionation as no further metabolic processing is required, thereby maintaining the relatively low δ^13^C values associated with lipids as they are incorporated into the parasite. The relatively uniform neutral or negative relationships for Δ^13^C values across the pairings located from both the dietary tract and the general cavity indicate that lipid carbon is likely utilized to support tissue growth beyond species closely associated with fatty tissues (e.g. blood and liver)^13,14^. This relationship should be examined further with methods that incorporate metabolic pathway techniques for target species beyond model organisms targeted for pathogenicity^24,25^. Pairings that have elevated Δ^13^C values likely reflect decreased utilization of host lipids and increased reliance on either host sugars or proteins processed through more complete metabolic pathways within the helminths to provide tissue carbon or potentially different δ^13^C compositions from different host tissues^18^.

### Comparing host isotopic values and Δ^13^C and Δ^15^N of parasite–host pairings

δ^15^N values for both fish host species *L. genivattus* and *N. furcosus* increased with body size leading to higher trophic levels in larger fish, a pattern commonly observed for coral reef-associated invertivore/carnivore fish, while there was no corresponding increase or shift in δ^13^C throughout ontogeny. No change in δ^13^C indicates that both smaller and larger individuals are relying on similar sources of underlying carbon production^33^, and that the larger individuals are feeding on larger prey with an elevated trophic level, i.e. elevated δ^15^N values^34^.

Additionally, a negative linear relationship was observed for both Δ^13^C and Δ^15^N values *versus* δ^13^C and δ^15^N values for predatory fish hosts were observed with significant negative slopes for nitrogen in 7 of the 13 parasite–host pairs (Fig. 4; Supplementary Table 2), while no significant within-pairings relationships were found for carbon (Supplementary Table 2). A negative linear relationship for trophic discrimination factors with increased host carbon and nitrogen values has previously been observed for parasite–host, predator–prey and herbivore-plant relationships^12,14,35^ and may be associated with dietary quality. In this study, there appeared to be an increased spread in the fractionation (Δ^15^N values) observed within the herbivore *S. lineatus*, with the lowest δ^15^N values occurring with the highest Δ^15^N values and a distinct grouping of individuals with lower Δ^15^N values corresponding with the highest δ^15^N values (Fig. 4). This wide range of values may represent the relative richness in diet, with strictly herbivorous individuals causing a shift in their parasites towards exclusive utilization of host tissues, while individuals that supplement with animal protein (more omnivorous) provide additional material within their diet for their parasites to supplement from. Increased protein quality in a predator’s diet results in a smaller ‰ difference between the diet and consumer, i.e. a smaller trophic fractionation^36^. This relationship coincides with the trend of decreased Δ^15^N values being observed for increased trophic level predation within predators in this study (Fig. 4). In herbivores, larger trophic discrimination factors for nitrogen are often observed^37^, and supplementation with protein (omnivory) would be expected to generate a negative offset in Δ^15^N if the parasites are supplementing from dietary protein in addition to host tissues.

## Conclusion

In conclusion, we found that positive discrimination for nitrogen occurred more often in dietary tract associated helminths, while neutral or negative discrimination occurred for helminths from within the general cavity. Increased discrimination in the dietary tract is likely due to a combination of the increased need for metabolism of food taken from the host’s diet and the host tissue in comparison to the helminths in the general cavity that appear to make use of direct uptake pathways for host nitrogen compounds with minimal metabolic reworking. No differences were observed between the two parasite habitats for discrimination of carbon. This study characterized discrimination factors for carbon and nitrogen within helminths living in coral reef fish and highlights the uncertainties that remain in adequately describing parasitic relationships within food webs. These uncertainties call for the development of a taxon- or species-specific and scaled framework for using bulk stable isotope analysis to study the trophic ecology of parasites. In addition, further work using metabolomics and compound specific stable isotope techniques is warranted to better characterize the underlying metabolic differences that are driving the differences observed for trophic discrimination factors between parasite and hosts.

## Methods

### Sampled areas and studied species

Individual fish from the three species *Lethrinus genivittatus* (Cuvier & Valenciennes, 1830), *Nemipterus furcosus* (Cuvier & Valenciennes, 1830), and *Saurida undosquamis* (Richardson, 1848) were caught using hand lines in the lagoon off the city of Nouméa (22°18′S and 166°25′E) in New Caledonia, southwestern Pacific Ocean at approximately 10–12 m depth, in August 2011, 2013, and 2014. Three years of data from catches were pooled as a preliminary two-way ANOVA (year × size) and revealed that year was not a significant factor (p > 0.05). *L. genivittatus* mostly feeds on crabs and worms, *N. furcosus* mostly feeds on crabs and shrimp and *S. undosquamis* is predominantly piscivorous^38^. The species *Siganus lineatus* (Cuvier & Valenciennes, 1835) was caught in coastal mangroves in the southeast coast at Yaté (22°16′S and 167°01′15E) using gillnets in June–August 2014. This species is usually considered an herbivore^39^, but has been observed to feed predominately on algae and to supplement with minor consumption of invertebrates in Yaté^40^. Parasites were present in all fish that were examined and appear to be ubiquitous within the species examined in this study.

All individuals caught were immediately placed in ice until further processing in the laboratory. Each fish was measured to the nearest 0.1 cm (total length) and a small piece of dorsal muscle of each fish was sampled and immediately frozen at − 20 °C for further stable isotope analyses. To extract the parasites, the general cavity was first examined to collect parasites embedded in or attached to fish tissues outside of the digestive tract. In a second step, the method presented in Justine et al.^41^ was applied to flush and extract living parasites from within the digestive tract using a 9% saline solution that was then briefly brought to near boiling to fix the parasites prior to transfer to 95% ethanol. All helminth parasites (i.e. nematodes, cestodes and trematodes) having a sufficient biomass were collected and immediately frozen. A total of 54 *L. genivittatus* were caught with 36 exploitable parasite-fish pairings, 99 *N. furcosus* with 75 exploitable parasite-fish pairings, and respectively 7 *S. undosquamis* and 18 *S. lineatus* were exploitable as parasite-fish pairings (Table 1). All animal experimentation met the ABS/ASAB guidelines for ethical treatment of animals and sampling protocols were approved by the internal ethics committee for the Université de la Nouvelle-Calédonie.

### Stable isotope preparation and analyses

Carbon and nitrogen stable isotope ratios (δ^13^C and δ^15^N) were determined for dorsal muscle tissue of all fishes collected. Fish muscle tissue is routinely utilized for stable isotope values for fish as it usually does not require lipid extraction prior to analysis^42^. Samples were freeze-dried and ground into a fine powder using a mortar and pestle. One milligram of powdered material was loaded into tin capsules and analyzed for each sample without prior treatment. This same procedure was used for parasites (whole animal) for samples that had sufficient dry mass (≥ 0.3 mg).

^13^C/^12^C and ^15^N/^14^N ratios were determined with a continuous-flow isotope ratio mass spectrometer (Thermo Scientific Delta V Advantage, Bremen, Germany) coupled to an elemental analyser (Thermo Scientific Flash EA1112, Bremen, Germany). The analytical precision was 0.1‰ for ^13^C and 0.15‰ for ^15^N, estimated using the internal standards leucine calibrated against ‘Europa flour’ and IAEA standards N1 and N2. Isotope ratios were expressed as δ notation (‰) differences from a standard reference material:

1 $$\updelta {\text{X }} = \, \left[ {\left( {{\text{R}}_{{{\text{sample}}}} /{\text{R}}_{{{\text{standard}}}} } \right) \, {-}{ 1}} \right] \, \times { 1}0^{{3}}; \quad {\text{ where X is}}\;^{{{13}}}{\text{C or}} \; ^{{{15}}}{\text{N}}$$

where R is the corresponding ratio (^13^C/^12^C or ^15^N/^14^N) for both sample and reference standard and δX is the measured isotopic value in per mil (‰) relative to the international standard references are Vienna Pee Dee Belemnite (vPDB) for carbon and atmospheric N_2_ for nitrogen.

Parasite–host discrimination factors were calculated using:

2 $$\Delta^{{{13}}} {\text{C or }}\Delta^{{{15}}} {\text{N }} = \updelta {\text{X}}_{{{\text{parasite}}}} {-} \, \updelta {\text{X}}_{{\text{host tissue}}}$$

where δX represents the isotopic value of carbon or nitrogen for each parasite–host tissue pairing examined.

### Data analysis

The significance of differences in δ^13^C and δ^15^N between a fish and its parasite was tested with the Wilcoxon signed rank test when homogeneity of variances was not verified or paired samples t-tests when homogeneity of variances was verified, dependant on fish species. The relationships between fish size and isotopic values (δ^15^N or δ^13^C) were investigated with Pearson correlation coefficients. One-way analysis of variance (ANOVA) was used to determine significant differences between host and parasite δ^13^C and δ^15^N values and to explore the relationship between host size and trophic level. The relationship between host δ^13^C and δ^15^N values and Δ^13^C and Δ^15^N values were determined through linear regression followed with subsequent application of an F test of the modelled slope against a slope of 0. This was done among and within the host-parasite pairings. For the analysis among pairings we excluded samples from the herbivorous fish host *S. lineatus* (18 samples) due to their very different isotope values to avoid skewing the relationship due to explained outliers.

The trophic level (TL) of fish individuals was calculated following the formulae of^10^:

3 $${\text{TL}} = \lambda + (\updelta^{{{15}}} {\text{N}}_{{{\text{fish}}}} {-}\updelta^{{{15}}} {\text{N}}_{{{\text{source}}}} ) \, /\Delta^{{{15}}} {\text{N}}$$

where λ is the trophic level of the source of organic matter, i.e. 1, : δ^15^N_fish_ is the isotopic value of nitrogen for the considered fish, δ^15^N_source_ is the isotopic value of the source of organic matter at the base of the food web, i.e. 3.59 for sedimentary organic matter^33^ that concerns *L. genivittatus*, *N. furcosus* and *S. undosquamis*, all caught of sandy unvegetated bottom; and 2.12 for the most eaten algae by *Siganus lineatus* and Δ^15^N that is the trophic enrichment factor (TEF) between a food item and its consumer. Here, we adopted a value of 3.9‰ for *S. lineatus*^40,43^ reflecting usually higher TEF for herbivores compared to the conventional 3.4‰ value^37^. For the three other species, we adopted a TEF of 3.0‰ because TEF are usually lower than the conventional value for carnivores^37,44^.

## Supplementary Information

Supplementary Information.
